# Comparison of Antibacterial Efficacy of Calcium Hydroxide Paste, 2% Chlorhexidine Gel and Turmeric Extract as an Intracanal Medicament and their Effect on Microhardness of Root Dentin: An *in vitro* Study

**DOI:** 10.5005/jp-journals-10005-1213

**Published:** 2013-10-14

**Authors:** AR Prabhakar, Swapnil Taur, Savita Hadakar, S Sugandhan

**Affiliations:** Professor and Head, Department of Pedodontics and Preventive Dentistry, Bapuji Dental College and Hospital, Davangere-577004 Karnataka, India, e-mail: attiguppeprabhakar@gmail.com; Assistant Professor, Department of Pedodontics and Preventive Dentistry, School of Dental Sciences, Krishna Institute of Medical Sciences Deemed University, Karad, Maharashtra, India; Assistant Professor, Department of Pedodontics and Preventive Dentistry, School of Dental Sciences, Krishna Institute of Medical Sciences Deemed University, Karad, Maharashtra, India; Professor, Department of Pedodontics and Preventive Dentistry, Bapuji Dental College and Hospital, Davangere, Karnataka, India

**Keywords:** Intracanal medicaments, Ca(OH)_2_, 2% Chlorhexidine gel, Turmeric extract, Microhardness

## Abstract

**Aim:** To evaluate and compare the antibacterial efficacy of turmeric extract as an intracanal medicament against *E. faecalis* and its effect on the microhardness of root dentin in comparison with calcium hydroxide and 2% chlorhexidine gel.

**Materials and methods:** One hundred and fourty dentin blocks were prepared from 70 extracted human single-rooted teeth and standardized. For antibacterial assessment, 120 blocks were infected for 21 days with *E. faecalis* (n = 24/group). Dentin blocks were treated with group I (Ca(OH)_2_), group II (2% chlorhexidine gel), group III (turmeric extract), group IV (saline) and group V (negative control). Dentin shavings were obtained in TSB at depth of 400 μm and plated to count CFUs at 24 hours, 3 and 7 days (n = 8/day). For microhardness assessment, eight samples of 2 mm thickness were prepared form four dentin blocks (n = 8/group)*.* Following treatment with medicaments, microhardness test was performed at 24 hours, 3 and 7 days using Vickers hardness indentation machine at 400 μm from canal lumen.

**Results:** Complete inhibition of *E. faecalis* was observed with group II, followed by 64% with group I and 54% with group III which was statistically highly significant (p < 0.001). Highest effect on microhardness of root dentin was shown by group I, followed by group II and no effect was seen with group III which was statistically highly significant (p < 0.001).

**Conclusion:** Turmeric extract has substantial antibacterial activity with no effect on microhardness of root dentine and hence has a potential to be used as intracanal medicament if its antibacterial activity could be enhanced.

**How to cite this article:** Prabhakar AR, Swapnil T, Savita H, Sugandhan S. Comparison of Antibacterial Efficacy of Calcium Hydroxide Paste, 2% Chlorhexidine Gel and Turmeric Extract as an Intracanal Medicament and their Effect on Microhardness of Root Dentin: An *in vitro* Study. Int J Clin Pediatr Dent 2013;6(2):171-177.

## INTRODUCTION

The main purpose of root canal therapy is to eliminate microorganisms and their products from the root canal system as well as to prevent reinfection. In order to predictably eliminate as many bacteria as possible from the entire root canal system, a combination of mechanical instrumentation and irrigating solutions has been used to remove or dissolve organic debris and to destroy bacteria. Interappointment intracanal medication has been unequivocally shown to contribute to favorable outcomes when treating persistent endodontic infections.^1^

Calcium hydroxide [Ca(OH)_2_] is the most commonly used endodontic medicament and eliminates most microorganisms due to high pH (12.8) when used as a 7-day dressing. However, it is unable to kill *Enterococcus faecalis*, the most commonly isolated bacteria in failed endodontic cases.^2^

Chlorhexidine gluconate (CHX) has been recently used in endodontics as both an irrigant and as an intracanal medicament. It is a broad spectrum antimicrobial agent and is effective against bacterial strains which are resistant to (Ca(OH)_2_). CHX cannot be used as routine medicament as it lacks property of tissue solubility and possesses cytotoxic effect. Further, some individuals may also be allergic to it.^3^

In order to overcome the shortcomings of the present medicaments, herbal plant extracts can be used. In endodontics, recent trend attends to use biologic medication extracted from natural plants.^4^

Turmeric (*Curcumin longa*) has been used for 1000 of years as a medicinal herb. The Curcumin, a phenolic compound has shown bactericidal properties by clinical testing with a greater medicinal effect like antioxidant, anti-inflammatory, antimicrobial, antispasmodic, anticancer and many other properties which may prove to be a boon to dentistry. This study intended to explore the antibacterial property of Curcumin for its use as an intracanal medicament owing to its antibacterial properties, ease of availability, low cost and lack of adverse effects.^5^

It has been reported that some chemicals used during endodontic procedures are capable of causing surface alterations of dentin which in turn cause reduction in microhardness rendering dentin structurally nonsupportive and this may affect the final restored tooth.^6,7^

Keeping these above concepts in mind, this study was designed to explore a new material, turmeric extract not only as an intracanal medicament but also to evaluate its effect on the microhardness of root dentin in comparison with calcium hydroxide and 2% chlorhexidine gel.

## MATERIALS AND METHODS

### Preparation of Medicament

- Ca (OH)_2_ powder was mixed with sterile saline in the ratio of 1.5:1 (wt/vol) to obtain a paste.^8^
- 2% CHX gel was prepared by mixing 2% CHX gluconate solution of pH 7.0 and 8 gm of water soluble gel base, hydroxyethyl cellulose (1% natrosol).^3^
- Aqueous turmeric extract: 200 gm ground turmeric powder was dried in oven at 40°C for 24 hours. After which it was boiled in 500 ml of distilled water and spray dried to make a paste. pH of extract was adjusted to 6.5 with aqueous buffers.^9,10^

The experimental groups (n = 24) were divided as group I (Ca(OH)_2_), group II (2% CHX gel), group III (turmeric extract), group IV (saline-positive control) and group V (negative control).

### Preparation of Dentin Blocks

Seventy single-rooted mature permanent human teeth extracted for therapeutic reasons were selected for this study. Model proposed by Haapasalo and Orstavik was modified for preparation of dentine blocks. One hundred and fourty dentin blocks were prepared such that two dentin blocks were made from one single tooth. Standardization of blocks was done (6 mm height and internal diameter standardization with ISO 012 bur was done and cementum was removed. Blocks were treated with 17% ethylenediaminetetraacetic acid followed by 5% sodium hypochlorite to remove organic debris. Sterilization of dentin blocks were done by autoclaving at 121°C.^11^ Twenty dentin blocks were randomly selected for microhardness test and remaining 120 blocks were used for antibacterial assessment.

### Contamination of Blocks

*E. faecalis* (ATCC 29212) was used as the test organism cultured in Tryptone Soya Broth (TSB). Bacterial inoculum was standardized to 0.5 McFarland turbidity standard. Dentin blocks were infected with 2 ml TSB containing 50 μl of *E. faecalis* under laminar flow. Incubation of blocks was done at 37°C for 21days during which broth was changed every 2 days. Medicaments were incorporated in respective dentine blocks and incubated.^11^

**Fig. 1:**
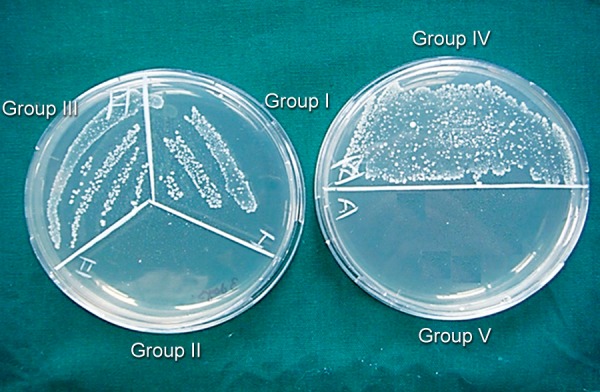
CFUs in test groups and control groups at the end of 24 hours

**Fig. 2:**
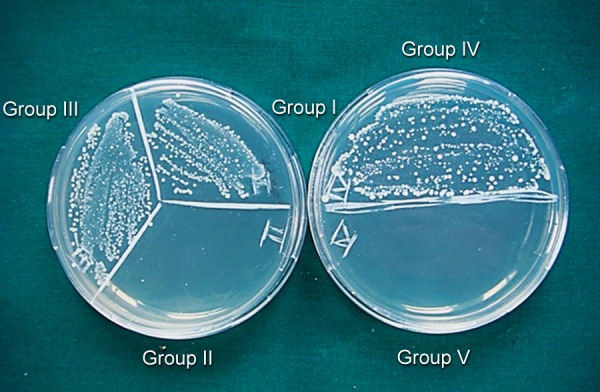
CFUs in test groups and control groups at the end of 3 days

**Fig. 3:**
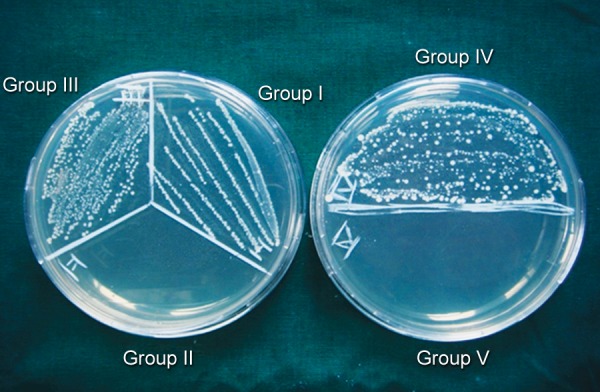
CFUs in test groups and control groups at the end of 7 days

Following treatment with medicament, dentin blocks were sealed with paraffin wax at both ends. Antibacterial assessment was done at the end of 24 hours (Fig. 1), 3 and (Fig. 2) 7 days (Fig. 3). After removal of medicament with saline, the dentin shavings were harvested at the depth of 400 μm (ISO 016 bur) from eight specimens of each group and collected in 1 ml of TSB. This TSB was serially diluted and plated on TS agar, incubated for 24 hours and colony-forming units (CFUs) were counted.^11^

### Root Dentin Microhardness Assessment

**Fig. 4:**
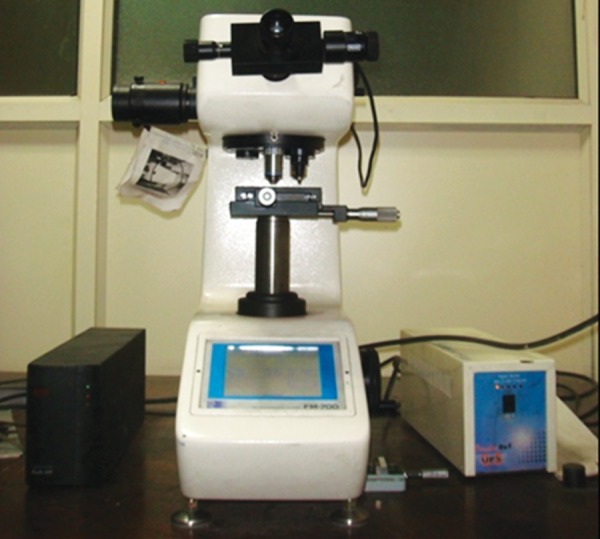
Vickers hardness tester (Future-Tech Corp FM-700, Tokyo, Japan)

**Fig. 5:**
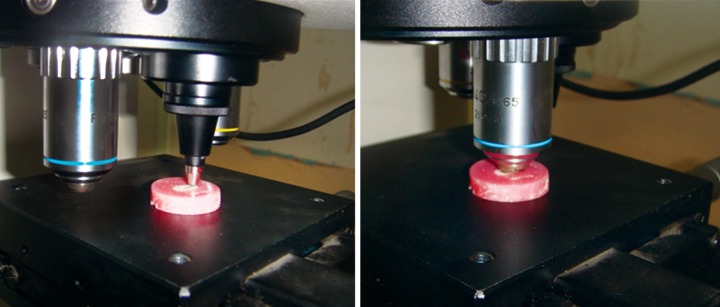
Microhardness testing of dentin samples

**Fig. 6:**
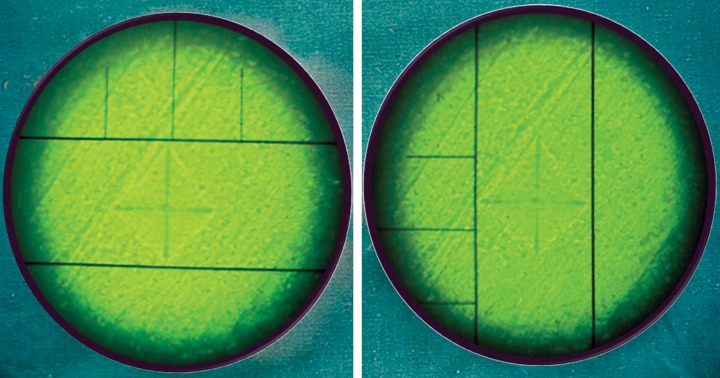
Microscopic view of indentation and measurement of VHN

Eight specimens were prepared form four dentin blocks and embedded in acrylic resins to make 2 mm dentin disk. Specimens were polished with abrasive papers (Matador abrasive papers 350, Germany). These specimens were kept in the airtight containers saturated with respective medicaments. Following treatment with medicaments, microhardness test of dentin blocks was done after 24 hours, 3 and 7 days using Vickers hardness indentation machine (Fig. 4) (Future-Tech Corp FM-700, Tokyo, Japan) at 400 μm from canal lumen. Baseline data was recorded from positive and negative control groups. All the indentations were made with 200 gm and dwell time of 15 seconds. Dentin microhardness was measured at three different points (Figs 5 and 6) and mean was calculated. The values were recorded as Vickers hardness number (VHN).^6^ The data was recorded, tabulated and statistically analyzed.

## STATISTICAL ANALYSES

### Antibacterial Activity

Friedman's test and Wilcoxon's signed-rank test for intragroup comparison. Kruskal-Wallis test and Mann-Whitney U test for intergroup comparison were done.

### Microhardness Test

Tukey's post hoc test for time dependent intragroup comparison and one-way ANOVA for intergroup comparison were done.

## RESULTS

### Antibacterial Activity

**Graph 1:**
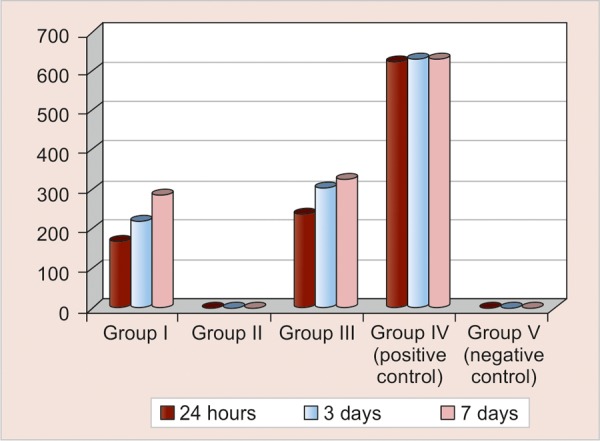
Comparison of bacterial counts (CFUs) in group III at the end of 24 hours, 3 and 7 days

Table 1 and Graph 1 show the intergroup comparison of antibacterial activity (CFUs) at 24 hours, 3 and 7 days. Group II (2% CHX gel) showed complete inhibition of bacterial growth on all days and was most effective against *E. faecalis* followed by group I [Ca(OH)_2_] and group III (turmeric extract). These groups were statistically highly significant (p < 0.001). On pair-wise comparison, all test groups showed statistically significant difference at the end of 24 hours, 3 and 7 days.

Table 2 shows the intragroup comparison of antibacterial activity at 24 hours, 3 and 7 days. Group II showed complete inhibition of bacterial growth at all days, while groups I and III showed decreased antibacterial activity at 3 days which further decreased at 7 days. Group I showed statistically high significant difference (p < 0.001) while group III showed significant difference (p = 0.008) between 24 hours and 3 days and 24 hours and 7 days respectively.

### Microhardness Assessment

Table 3 shows the intragroup comparison of microhardness values (VHN) at the end of 24 hours, 3 and 7 days. Group III showed no effect on microhardness of root dentin at all days, while groups I and II showed decreased microhardness at 3 days that further decreased at the end of 7 days which was statistically highly significant (p < 0.001). Groups I and II showed statistically significant difference between 24 hours and 7 days and 3 and 7 days respectively.

**Graph 2:**
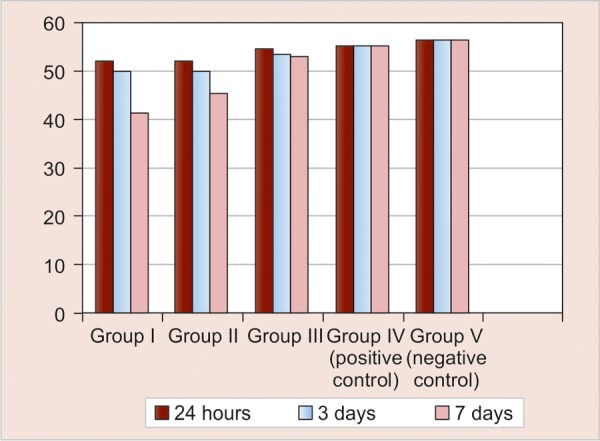
Comparison of microhardness values (VHN) among different groups at the end of 24 hours, 3 and 7 days

Table 4 and Graph 2 show the intergroup comparison of microhardness values (VHN) at the 24 hours, 3 and 7 days. The maximum decrease in microhardness (VHN) of root dentin was observed in group I followed by group II while group III had no effect.

To summarize, the present study results stated that on an average 100% inhibition of *E. faecalis* at 400 μm depth was observed with 2% CHX gel, followed by 64% with Ca(OH)_2_ and finally 54% with turmeric extract. Highest decrease in microhardness was shown by Ca(OH)_2_, followed by 2% CHX gel and no effect of turmeric extract.

**Table 1:** **Table 1:** Descriptive statistics showing the intergroup comparison of bacterial counts (CFUs) among different groups at the end of 24 hours, 3 days and 7 days

*Time of assessment*	*Groups with mean and SD*									p*-value, sig		Pairwise comparison**
	*Group I*		*Group II*		*Group III*		*Group IV*		*Group V*			
*24 hours*	*170 ± 16.0*		*0.0 ± 0.0*		*238.1 ± 26.8*		*626.9 ± 68.6*		*0.0 ± 0.0*	<0.001 HS		I & III(68.0)
												I & IV(456.8)
												III & IV(378.5)
3 days	*221.3 ± 27.4*		*0.0 ± 0.0*		*306.1 ± 25.0*		*632.4 ± 53.8*		*0.0 ± 0.0*	<0.001 HS		I & III(84.9)
												I & IV(411.1)
												III & IV(326.3)
7 days	*285.4 ± 19.6*		*0.0 ± 0.0*		*328.4 ± 23.0*		*631.3 ± 41.6*		*0.0 ± 0.0*	<0.001 HS		I & III(65.5)
												I & IV(444.0)
												III & IV(378.5)

* Kruskal-Wallis test; **Mann-Whitney U test; HS: Highly significant

**Table 2:** **Table 2:** Descriptive statistics showing the intragroup comparison of bacterial counts (CFUs) in different groups at the end of 24 hours, 3 days and 7 days

*Groups with mean and SD*		*Time of assessment*						p*-value, sig		Pairwise comparison**
		*24 hours*		*3 days*		*7 days*				
Group I		170.1 ± 16.0		221.3 ±27.4		285.4 ± 19.6		<0.001 HS		24 hours & 3 days (51.1),
										24 hours & 7 days (115.3)
Group II		0.0 ± 0.0		0.0 ± 0.0		0.0 ± 0.0		–		–
Group III		238.1 ± 26.8		306.1 ± 25.0		328.4 ± 23.0		0.008 S		24 hours & 3 days (68.0),
										24 hours & 7 days (90.3),
Group IV		626.9 ± 68.6		632.4 ± 53.8		631.3 ±41.6		0.2 NS		–
Group V		0.0 ± 0.0		0.0 ± 0.0		0.0 ± 0.0		–		–

*Friedman's test; **Wilcoxon's signed-rank test; HS: Highly significant, S: Significant; NS: Not significant

**Table 3:** **Table 3:** Descriptive statistics showing the intragroup comparison of microhardness values (VHN) in different experimental groups at the end of 24 hours, 3 and 7 days

*Groups with mean and SD*		*Time of assessment*						p*-value, sig		Significant pairs**
		*24 hours*		*3 days*		*7 days*				
Group I		52.03 ± 1.23		49.85 ± 2.16		41.51 ± 2.37		<0.001 HS		24 hours & 7 days (10.53),
										3 days & 7 days (8.35)	
Group II		52.14 ± 2.22		50.04 ± 1.65		45.44 ± 2.54		<0.001 HS		24 hours & 7 days (6.7),	
										3 days & 7 days (4.6)	
Group III		54.67 ± 1.63		53.43 ± 1.57		53.00 ± 0.85		0.07 NS		–	

*One-way ANOVA test; **Tukey's post hoc test; HS: Highly significant; NS: Not significant

**Table 4:** **Table 4:** Descriptive statistics showing the intergroup comparison of microhardness values (VHN) among different groups at the end of 24 hours, 3 and 7 Days

*Time of assessment*		*Groups with mean and SD*										p*-value, sig		Pairwise comparison**
		*Group I*		*Group II*		*Group III*		*Group IV (positive control)*		*Group V* *(negative* *control)*				
24 hours		52.03 ± 1.23		52.14 ± 2.22		54.67 ± 1.63		55.06 ± 3.36		56.28 ± 2.68		0.002 S		I & V, II & V
3 days		49.85 ± 2.16		50.04 ± 1.65		53.43 ± 1.57		55.06 ± 3.36		56.28 ± 2.68		<0.001 HS		I & III, I & IV,
														II & IV, I & V,
														II & V
7 days		41.51 ± 2.37		45.44 ± 2.54		53.00 ± 0.85		55.06 ± 3.36		56.28 ± 2.68		<0.001 HS		I & II, I & III
														II & III, I & IV
														II & IV, I & V,
														II & V, III & V

*Oneway ANOVA; test

** Tukey's post hoc test; S: Significant; HS: Highly significant

## DISCUSSION

### Antibacterial Assessment

Biomechanical preparation alone is not capable of thoroughly eliminating microorganisms from the complex root canal system. Investigators have noted that bacteria in instrumented, unfilled canals can multiply and reach their pretreatment numbers in 2 to 4 days. In such cases, dressing of root canals using antimicrobial medicaments are advocated *E. faecalis* is a resistant microorganism that plays an important role in persistent periapical lesions.^1^

This study was a modest attempt to evaluate and compare the antibacterial efficacy of calcium hydroxide paste, 2% chlorhexidine gel and aqueous turmeric extract in dentin blocks infected by *E. faecalis* and their effect on microhardness of root dentin.

The present study revealed that highest antibacterial activity was observed with group II (2% CHX gel). The plausible reasons could be the high concentration of CHX (2%), lethal bactericidal mode of action and enhanced diffusion into dentinal tubules as CHX gel has low contact angle with dentin and thus penetrates the dentinal tubules effectively at faster rate.^3,11^

Portenier et al stated that dentin matrix and type I collagen have inhibitory effect on chlorhexidine, but these studies tested a concentration of 0.2% CHX. The inhibitory effect of dentin on CHX can be overcome by increase in concentration (2%) as used in this study and achieved complete inhibition.^12^

Antibacterial activity of Ca(OH)_2_ can be attributed to direct contact through high pH (12.5-12.8) and its ability to dissociate into hydroxyl ions causing bacterial cell death.^2^Ca(OH)_2_ showed decreased antibacterial activity over a period of time which may be attributed to several factors.

Firstly, the buffering of the alkalinity of Ca(OH)_2_ by dentin and dentin components. Secondly, low diffusibility of hydroxyl ions in dentinal tubules. Thirdly, *E. faecalis* colonize within dentinal tubules forming dense biofilms, such that bacteria located within the dentinal tubules can protect those located deeper inside the tubules thus evading the hydroxyl ions. Evans et al demonstrated that the proton pump activity of *E. faecalis* offers resistance to high pH of calcium hydroxide.^8,11^

Turmeric extract had substantial antibacterial effect as compared to positive control. This effect can be attributed to antibacterial action of ingredients of turmeric that are responsible for its biologic activity which is Curcumin. The aqueous extract containing Curcumin as the main ingredient.^9^ It is suggested that Curcumin, a polyphenolic compound, strongly inhibits bacterial cell proliferation by inhibiting the assembly dynamics of FtsZ in the Z-ring needed for bacterial cell division. Curcumin has been shown to have a potent antibacterial activity against a number of pathogenic bacteria including Enterococcus.^13^

However, there was a gradual decrease in antibacterial activity of the turmeric extract at 3 and 7 days. This decreased effect can be due to buffering ability of dentin which may affect activity of Curcumin. Curcumin is stable at a pH of 6.5 but highly unstable at neutral-basic pH conditions.^10^ Even slight increase in pH will significantly affect the activity of Curcumin and thus rendering it ineffective against *E. faecalis* in dentinal tubules. Thus, this might be a contributing factor for its decrease in antibacterial efficacy over a period of time.

Although the present study has shown CHX to be the most effective antibacterial agent, concern over the possible cytotoxic effects has shifted the focus onto turmeric extract. This study confirms and supports the use of turmeric (*Curcumin longa*) for antimicrobial treatment of root canal infection. The findings of the present study may help us to design more potent Curcumin analogs with improved stability. Thus, further studies in this field are required so as to enhance the antibacterial activity of turmeric.

### Microhardness Assessment

It has been stated that microhardness determination can provide indirect evidence of mineral loss or gain in dental hard tissues as it depends on the amount of calcified matrix per square millimeter. The relative softening effect exerted by intracanal irrigant and medicament on the dentinal walls could affect the adhesion and sealing ability of sealers to the treated dentin surfaces.^14^ Therefore, this parameter was selected in the current study.

Vickers microhardness testing was employed as it is more sensitive to measurement errors, less sensitive to surface conditions and small specimens can be tested with good accuracy.^15^

The observations showed that group III (turmeric extract) had the least effect on microhardness followed by group II (2% CHX gel) and group I [Ca(OH)_2_]. The microhardness values reduced over a period of time for groups I and II which was statistically highly significant, whereas group III showed no reduction in microhardness.

Yoldas et al stated that Ca(OH)_2_ showed decrease in microhardness which could be due to the proteolytic action of Ca(OH)_2_. The pH increase observed after exposure to Ca(OH)_2_ may reduce the organic support of the dentin matrix causing breakdown of protein structure and disruption in links between the collagen fibers and the hydroxyapatite crystals that could negatively influence the mechanical properties of dentin.^6^

This study used 2% CHX gel formulation which is not tested in previous studies when used as intracanal medicament for 7 days. Interestingly, 2% CHX gel showed moderate reduction in the microhardness values (VHN). Similar observation was made by Oliveira et al, where 2% chlorhexidine solution when used as an irrigant for 15 minutes significantly decreased the microhardness of root canal dentin.^7^ Thus, this unique observation in our study can be attributed to the significant alteration in dentin following treatment with high concentration (2% CHX) indicating potent direct effects of this chemical on the components of dentin structure disrupting the links between collagen fibers and hydroxyapatite crystals causing decrease in dentin microhardness which was time dependent. In addition, Ferraz et al, in a clinical study, showed that 2% CHX gel produced a cleaner and smoother root canal surface thus suggesting that it had some softening effect on root dentin thus supporting our observation.^3^

Interestingly, turmeric extract showed no effect on microhardness (VHN). Literature lacks evidence in reference to plant extract having any effect on microhardness of root dentin. From the observation made in the present study, it can be stated that as turmeric being a plant extract shows no effect on microhardness of root dentin over a period of time. It is recommended that long-term evaluation of turmeric extract should be carried in order to confirm this observation by histologic and chemical analysis.

This study recommends the use of turmeric (*Curcumin longa*) for antimicrobial treatment of root canal infection without affecting microhardness of root dentin. Before extrapolating, this result to clinical situations, it has to be evaluated for other properties like biocompatibility, staining and substantivity which are required for its use as an efficient intracanal medicament. Therefore, further studies are required in this area to prove it as a novel medicament.

## CONCLUSION

1. Two percent CHX gel had most effective antibacterial activity among the medicaments tested, but had adverse effect on microhardness of root dentin, so its clinical application may be cautioned.
2. Calcium hydroxide had moderate antibacterial activity with most deteriorating effect on microhardness of root dentin, hence its use as an intracanal medicament for shorter duration alone is appreciable.
3. Aqueous turmeric extract had substantial antibacterial activity with no effect on microhardness of root dentin. Hence, it has a potential to be used as intracanal medicament if its antibacterial activity could be enhanced.

The results of this study are encouraging and it can be conclude that turmeric (*Curcumin longa*) can be used as an antibacterial agent in the treatment of infected root canal with added advantages of ease of availability, cost effectiveness and other biological activities.
